# High-retention sodium supercapacitors with sodium hexametaphosphate-controlled water-processable/non-flammable sodium-ion solid-state electrolytes

**DOI:** 10.1038/s41378-026-01191-7

**Published:** 2026-02-11

**Authors:** Deepu Murukadas, Dahyeon Park, Minjae Kim, Hwajeong Kim, Youngkyoo Kim

**Affiliations:** 1 Organic Nanoelectronics Laboratory and KNU Institute for Nanophotonics Applications (KINPA), Department of Chemical Engineering, School of Chemical Engineering and Applied Chemistry, Kyungpook National University, Daegu, 41566 Republic of Korea; 2 Department of Energy Convergence & Climate Change and the Institute for Global Climate Change and Energy, Kyungpook National University, Daegu, 41566 Republic of Korea; 3 School of Semiconductor Convergence Engineering, Kyungpook National University, Daegu, 41566 Republic of Korea; 4 Priority Research Center, Research Institute of Environmental Science & Technology, Kyungpook National University, Daegu, 41566 Republic of Korea

**Keywords:** Organic-inorganic nanostructures, Electrical and electronic engineering

## Abstract

Achieving high-performance sodium-based solid-state electrolytes (SSEs) through environmentally friendly processes is crucial to establishing a solid foundation for safe and inexpensive energy storage devices. Here we demonstrate nonflammable sodium cation-transporting SSEs prepared from aqueous solutions of branched poly(ethylene imine) (bPEI), sodium hydroxide (NaOH), and sodium hexametaphosphate (SHMP). The bPEI:NaOH:SHMP (PNaS) SSEs exhibited an outstanding ion conductivity of ~1 mS/cm at SHMP = 20 mol%, which is 5 times higher than 0.18 mS/cm for the bPEI:NaOH (PNa) SSEs, due to the SHMP-induced morphology optimization for efficient Na^+^ transport. The optimum PNaS SSEs could deliver the output voltage of 4.4 V by galvanostatic charging at 0.5 mA/g, exhibiting long-term retention characteristics (>1000 s). The PNaS supercapacitors exhibited stable operation with 99.68% capacitance retained during 2000 charging/discharging cycles, while the PNaS films were considerably stable without burning upon the flammability test.

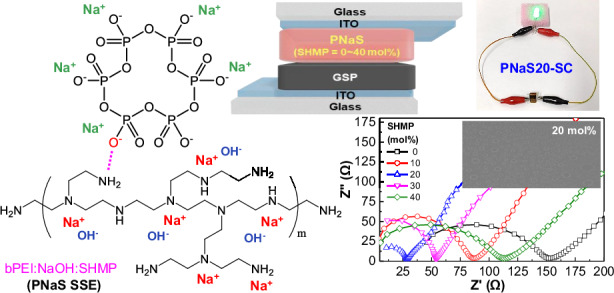

High demand for efficient, safe, and sustainable energy storage systems (ESSs) has driven extensive research into innovative materials and device architectures^1–7^. Currently, secondary batteries and supercapacitors with lithium-ion-based liquid electrolytes have been commercially positioned as top-notch due to their high energy and/or power densities enabled by the unrivaled electrochemical potential of lithium compared to other elements^8–13^. Despite such high performances, the flammability issue of the liquid electrolytes gradually freezes further spreading of lithium-ion batteries and supercapacitors for the ESS applications^14–18^.

On this account, keen attention has been paid to solid-state electrolytes (SSEs), which do not contain flammable liquids and thus potentially lead to safe ESSs^19–25^. In particular, polymer-based SSEs have been highlighted owing to their advantages of solution-based low-temperature processes and flexible/lightweight features^26,27^. Recently, in step with this benefit, aqueous solution-based processes for polymeric SSEs have been attempted by employing water-soluble polymers to fabricate environmentally friendly supercapacitors (see Table S1)^28–40^. However, most previous studies on water-processable polymeric SSEs utilized lithium-containing chemicals (salts) to generate lithium cations in the polymer matrix^38,39,41^.

Compared to lithium-based ESSs, sodium-based ESSs have the strong benefit of low-cost fabrication due to the natural abundance of sodium from sea salts, etc^42–44^. Hence, the sodium-based electrolytes have attracted intense interest even though the relatively larger ionic radius of sodium cations than lithium cations remains to be overcome^45,46^. However, most previous studies have employed halogenated sodium salts such as sodium bromate (NaBrO_3_) and sodium hypochlorite (NaClO) for water-based processes of SSEs^47,48^. To comply with the *Treaty on the Functioning of the European Union* (TFEU) and *UN Transpor*t guidelines, regulating hazardous halogen chemicals^49–51^, non-halogen sodium-containing compounds are required to prepare water-processable sodium-based polymeric SSEs.

In this work, we demonstrate water-processable sodium-based polymeric SSEs prepared using ternary mixtures of branched poly(ethylene imine) (bPEI), sodium hydroxide (NaOH), and sodium hexametaphosphate (SHMP). Here, SHMP, a cyclic ionic compound, was introduced as a sodium cation-containing additive due to its key features of high water solubility, low toxicity, and non-halogen compound^52,53^. For systematic investigation, the molar ratio of SHMP to the bPEI repeating unit was gradually varied up to 40 mol%. The resulting bPEI:NaOH:SHMP (PNaS) SSEs exhibited a high ion conductivity of ~1 mS/cm, enabling durable asymmetric sodium supercapacitors with a single-electrode-material geometry composed of graphite-based anodes between two current-collecting electrodes of indium tin oxide (ITO). The optimized PNaS-based sodium supercapacitors exhibited an output voltage of 4.1 V upon charging at 0.5 mA/g and excellent stability (99.68% capacity retention) after 2000 charge/discharge cycles.

## Aqueous processes for sodium solid-state electrolytes

**Fig. 1:**
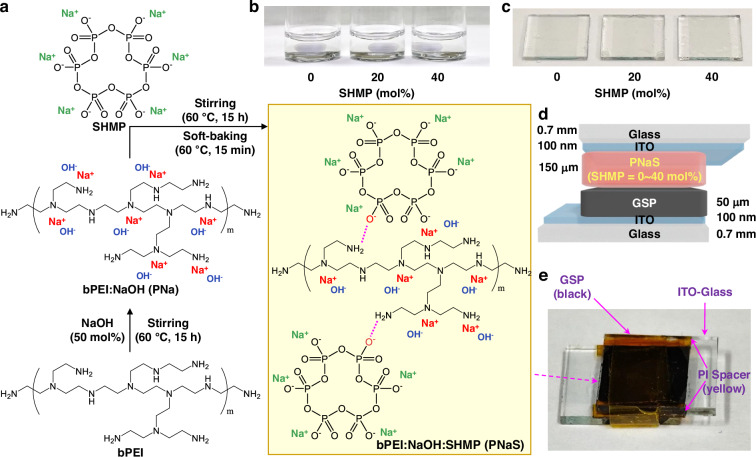
Solutions/films and device structure. **a** Schematic illustration for the preparation of bPEI:NaOH:SHMP (PNaS) SSEs from aqueous solutions of bPEI:NaOH (PNa) by the addition of SHMP. **b** Photographs for the aqueous ternary solutions of PNaS according to the SHMP molar ratio. **c** Photographs for the PNaS films according to the SHMP molar ratio. **d** Illustration on the cross-section of an asymmetric supercapacitor with the PNaS SSEs. **e** Photograph of the sodium supercapacitors fabricated by sandwiching the PNaS-coated GSP/ITO-glass electrode (top) and the PNaS-coated ITO-glass electrode (bottom)

First, aqueous solutions of binary component electrolytes (bPEI:NaOH, abbreviated as PNa) were prepared by mixing NaOH and bPEI at a fixed NaOH molar ratio of 50 mol% relative to the repeating unit of bPEI polymer (Fig. 1a). As shown in Fig. 1b, the bPEI:NaOH solutions were optically clear without any traceable solids. As NaOH dissociates into Na^+^ and OH^-^ in water, the sodium cations could make charge interaction with lone pair electrons of nitrogen atoms in bPEI so that they form **Na**^**+**^**---:N** structures (see the scheme in the middle of Fig.1a)^54^. To these binary (bPEI:NaOH) solutions, various amounts of SHMP (10, 20, 30, 40, 50 mol% relative to the bPEI repeating unit) were added to make ternary mixture solutions of bPEI:NaOH:SHMP (PNaS). All the ternary solutions were still optically clear (Fig. 1b), indicating excellent dispersion (dissolution) of all components in the aqueous state.

Considering that sodium cations are dissociated from SHMP in water, the resulting cyclic phosphate anions might interact with the H-N groups in bPEI by the dipole of the O = P groups. In addition, the SHMP anions could be additional binding (or hopping) sites for the sodium cations from NaOH. As observed in Fig. 1c, the PNaS films coated on ITO-coated glasses were optically transparent and colorless without any aggregates. This result indicates that the three components are well dispersed on a nanoscale in the solid state. Based on the film coating conditions, asymmetric sodium supercapacitors were fabricated by sandwiching the PNaS SSE films between the cathode-free ITO-glass and the anode-coated ITO-glass (note that the anode layers were prepared from the mixture of graphite, super P–Li, and poly(vinylidene fluoride) (PVdF), abbreviated as GSP) (Fig. 1d) (see the Methods section for details)^55^. The sodium supercapacitors with the PNaS SSEs were tightly wrapped using a polyimide (PI) film tape for secured contacts, while the thickness (150 μm) of PNaS SSE layers was controlled by the PI spaces on the edge (Fig. 1e).

## Ion conductivity and electrochemical characteristics

**Fig. 2:**
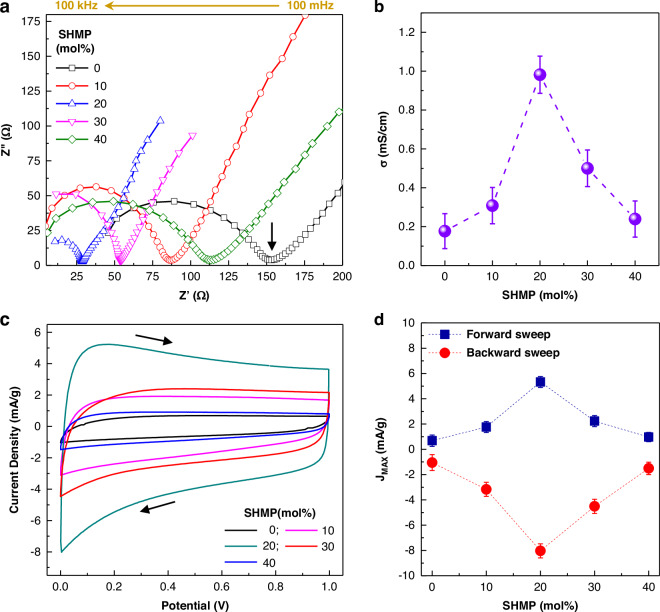
Ion conductivity and electrochemical characteristics. **a** Nyquist plots of glass/ITO/GSP/PNaS/ITO/glass devices according to the SHMP molar ratio (0–40 mol%) (frequency sweep from 100 kHz to 100 mHz). **b** Ion conductivity (**σ**) of the PNaS SSEs as a function of SHMP molar ratio. **c** Cyclic voltammetry (CV) curves of the supercapacitors with the PNaS SSEs at a sweep rate of 1.0 V/s (arrows denote the direction of potential sweeps). **d** Maximum current density (J_MAX_) at the forward and backward sweeps from the CV curves in (**c**)

As shown in Fig. 2a, all the Nyquist plots of the PNaS SSE films deliver semi-circles irrespective of the SHMP molar ratio, indicative of ionic charge transport in films^56^. A close look into the Nyquist plots finds that the real-part intercept of semi-circles, corresponding to the charge transfer resistance, is located at a lower impedance for the PNaS SSEs than the binary PNa SSEs. This means that the ion conductivity of the SSE films was improved by the addition of SHMP to the PNa SSEs (see the caption of Fig. 2 for the relation between ion conductivity and charge transfer resistance). The ion conductivity initially increased at SHMP = 10 mol%, reached a maximum (~1 mS/cm) at 20 mol%, and gradually decreased at higher SHMP contents (~40 mol%) (Fig. 2b). Here, it is worth noting that the ionic conductivity of ~1 mS/cm is included among the one of the highest values reported for polymer-based SSEs (Supplementary Table 1)^28–40^.

To clarify whether the residual water in the electrolyte films affected such a high ion conductivity, electrochemical impedance spectroscopy (EIS) measurements were conducted for the SSE films dried for 30 days. The EIS result disclosed that the ion conductivity was marginally reduced from ~1 to 0.82 mS/cm after drying for 30 days (Supplementary Fig. 1). Therefore, it is briefly concluded that the remnant water merely influences the present high ion conductivity in the PNaS films. The high ion conductivity of the PNaS SSEs can be supported by their wider non-Faradaic windows of cyclic voltammetry (CV) curves, compared to that of the PNa SSEs (Fig. 2c), indicating the larger current hysteresis caused by the sodium ion transport in the PNaS SSEs. As summarized in Fig. 2d, the largest current hysteresis between the forward (charging) and backward (discharging) sweeps was obtained at SHMP = 20 mol%, which is about eight times larger than that at SHMP = 0 mol% and in good agreement with the EIS result (see Supplementary Fig. 2 for the compared CV curves between SHMP = 0 and 20 mol%). As observed in Supplementary Fig. 3 (0 V ~ 1 V) and Supplementary Fig. 4 (0 V ~ 4 V), the CV curves at low sweep rates exhibited Faradaic peaks at around 0.4 V and 0.7 V, indicating possible redox reactions in the materials (electrolytes and electrodes) of devices.

## Spectroscopic/morphological analysis and mechanism

**Fig. 3:**
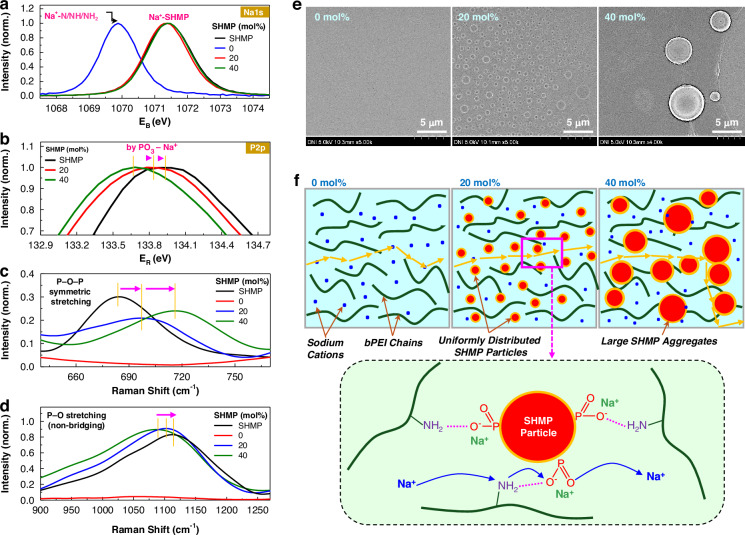
Spectroscopic and morphological analysis. XPS spectra for the pristine SHMP film and the PNaS films (**a**: Na1s, **b**: P2p). Raman spectra for the pristine SHMP film and the PNaS films: (**c**: symmetric stretching of P–O–P bonds, **d**: P–O stretching variations by non-bridged oxygen atoms. **e** FE-SEM images for the PNaS films according to the SHMP molar ratio. **f** Schematic representation for the proposed sodium-ion transport mechanism within the PNaS SSE films according to the SHMP molar ratio: The bottom panel illustrates molecular-level interactions between phosphate anions in SHMP particle and amine groups in bPEI chain, providing insight into the molecular-level Na^+^ (blue color) conduction within the PNaS matrix

To understand the enhanced ion conductivity by adding SHMP to the PNa SSEs, the molecular-level interactions in films were investigated using X-ray photoelectron spectroscopy (XPS). As shown in Fig. 3a, the Na1s peak observed at 1071.4 eV is attributed to interactions between sodium cations from NaOH and sodium hexametaphosphate (SHMP). An additional peak at a lower binding energy region (1069.8 eV) is assigned to sodium cations interacting with the polymeric chains in the absence of SHMP, indicating a change in the chemical environment surrounding the sodium ions. In particular, the close interaction between SHMP and bPEI can be explained by the shifted P2p peaks for the PNaS films, which is more pronounced at SHMP = 40 mol% (Fig. 3b). These interactions can be further supported by the Raman spectra (Fig. 3c), disclosing that the P-O-P symmetric stretching (700–800 cm^–1^) of SHMP is affected by the interaction with the bPEI chains in the PNaS films. In addition, the P–O stretching peak for the non-bridging units (1100–1150 cm^–1^) also gradually shifted with the SHMP content, which can be attributed mainly to the influence of SHMP-bPEI interactions leading to the environmental change of Na^+^ in both SHMP and PNa parts (Fig. 3d).

Based on the spectroscopic analysis results, the morphology of the PNaS films was investigated using field emission-scanning electron microscopy (FE-SEM). As shown in Fig. 3e, no particular morphological structure was measured from the surface of the PNa films. However, the PNaS films (SHMP = 20 mol%) showed an interesting morphology with randomly-distributed circular micro-domains (size = 0.1 – 3.0 μm). As the SHMP content increased to 40 mol%, the micro-domains grew bigger (size = 3 – 5 μm) and the film surfaces became considerably coarse. Based on the spectroscopic and morphological analysis results, a brief mechanism for sodium cation transport is proposed as illustrated in Fig. 3f: (1) At SHMP = 0 mol% (PNa SSEs), sodium cations transport by hopping along nitrogen atoms in bPEI chains so that the hydrophobic parts of bPEI chains can limit the Na^+^ transfer pathways; (2) At SHMP = 20 mol% (PNaS SSEs), the SHMP micro-domains make hydrophilic interfaces with the bPEI chains, enabling faster transfer of sodium cations under a reduced physical impedance by the hydrophobic part of bPEI chains see the enlarged illustration on the bottom part of Fig. 3f; (3) At SHMP = 40 mol% (PNaS SSEs), the effective hydrophilic pathways for Na^+^ transfer were destroyed by the formation of bigger SHMP micro-domains and coarse morphology, leading to the huge reduction in ion conductivity (see Supplementary Fig. 5 for the irregular and coarse morphology measured using the energy dispersive spectroscopy (EDS) system).

## Charging-discharging characteristics

**Fig. 4:**
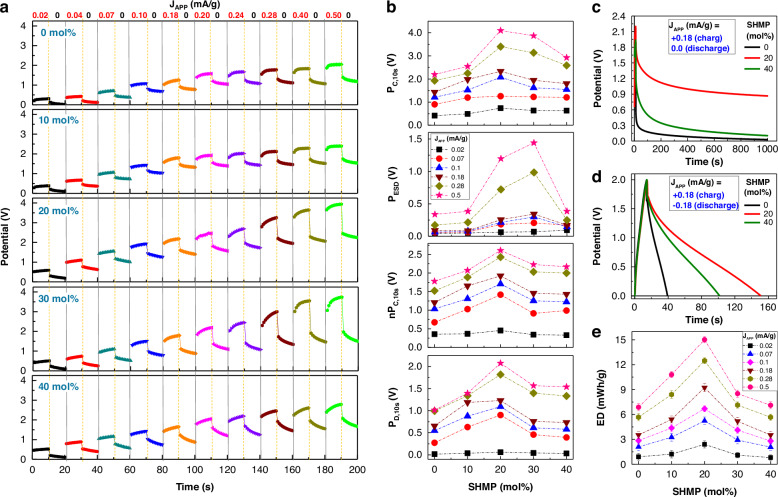
Charging/discharging characteristics. **a** Galvanostatic charge–discharge (GCD) curves of supercapacitors with the PNaS SSEs under the stepwise increase of applied current density (J_APP_) for charging (10 s each) and the natural discharging (J_APP_ = 0 mA/g). **b** The influence of SHMP molar ratio on the device potentials according to the varied J_APP_: (top) maximum potential charged for 10 s (P_C,10s_), (middle-top) potential drop by electrostatic discharge (P_ESD_), (middle-bottom) net maximum potential (nP_C,10s_) after subtracting P_ESD_ from P_C,10s_, (bottom) potential retained after discharging for 10 s after finishing charging step (P_D,10s_). **c** Long-term discharge behavior for the three representative SHMP molar ratios (natural discharge for 1000 s at J_APP_ = 0 mA/g after charging for 10 s at J_APP_ = 0.18 mA/g). **d** GCD curves under forced charge/discharge conditions by limiting the maximum potential to 2.0 V (charging at J_APP_ = +0.18 mA/g; discharging at J_APP_ = -0.18 mA/g). **e** Energy density (ED) as a function of SHMP molar ratio (see Supplementary Fig. 9 for the whole J_APP_ condition)

Next, the performance of sodium supercapacitors was evaluated using galvanostatic charge-discharge (GCD) methods. As shown in Fig. 4a, the initial charging step (10 s) at a current density (J_APP_) of 0.02 mA/g increased the potential (voltage) of all devices, ranging from 0.4 V to 4.1 V after 10 s, depending on the SHMP molar ratio. During the subsequent natural discharge phase (J_APP_ = 0 mA/g), the potential slowly faded out after an abrupt initial voltage drop due to electrostatic discharge (ESD) effects^57^. The stepwise increase of charging current density (up to 0.2 mA/g) resulted in gradually enhanced potentials depending on the SHMP molar ratio. These GCD data were analyzed to understand the trend of major parameters (Fig. 4b). The maximum apparent potential by charging at 10 s (P_C,10s_) was achieved at SHMP = 20 mol% over the entire J_APP_ condition (see the top panel in Fig. 4b). However, as displayed in the middle-top panel of Fig. 4b, the ESD-induced potential (P_ESD_) was quite high depending on the SHMP molar ratio, which might be caused by the electrically insulating components (e.g., bPEI alkyl parts) enriched at the interfaces between the GSP surfaces and the PNaS SSE layers (Supplementary Fig. 6). Even after extracting the P_ESD_ contribution from the GCD data, the net potential by charging at 10 s (nP_C_,_10s_) reached the highest value (nP_C_,_10s_ = 2.6 V) at SHMP = 20 mol% (see the middle-bottom panel in Fig. 4b).

Interestingly, the potential retained after natural discharging (for 10 s), at a total elapsed time of 20 s from the initial charging (P_D,10s_), was still the highest at SHMP = 20 wt% under the whole J_APP_ condition. This result supports that the PNaS SSEs at SHMP = 20 wt% deliver an effective anode–electrolyte interface, keeping sodium cations well intercalated and trapped in the GSP parts, due to the optimized morphology that maximize the transfer of sodium cations (see the discussion in Fig. 3f). This is further confirmed by the long-term discharging test, as plotted in Fig. 4c, which demonstrates the higher potential level retained at SHMP = 20 wt% than at SHMP = 0 and 40 mol%, even after natural discharging for 1000 s (Supplementary Fig. 7 for the repeated long-term discharge behavior). Note that the discharging rate was noticeably slower at SHMP = 20 wt% than at SHMP = 0 and 40 mol% (Supplementary Fig. 8). The forced charge/discharge test, which limited the maximum potential to 2.0 V under J_APP_ = ± 0.18 mA/g, delivered the longest retention performance at SHMP = 20 mol% (Fig. 4d). As a consequence, the highest energy density of ca. 15 mWh/g (net energy density = 7.53 mWh/g after removing the ESD effect) was achieved for the PNaS-supercapacitors at SHMP = 20 mol%, even though the energy density was still higher or similar at the higher SHMP molar ratios (30 and 40 mol%) than SHMP = 0 mol% (PNa SSE) (Fig. 4e and Supplementary Fig. 9 for the whole J_APP_ conditions). Here, it is noted that a slightly low performance was measured for symmetric devices with the geometry of glass/ITO/GSP/PNaS/GSP/ITO/glass at SHMP = 20 mol% (see the GCD results in Supplementary Fig. 10ab). This can be attributed to the combined interfacial resistances at the two GSP electrodes of symmetric devices, compared to the single GSP electrode of asymmetric devices, which is supported by the lower current density observed in symmetric devices compared to asymmetric devices (see CV curves in Supplementary Fig. 10c).

## Long-term endurance and nonflammability

**Fig. 5:**
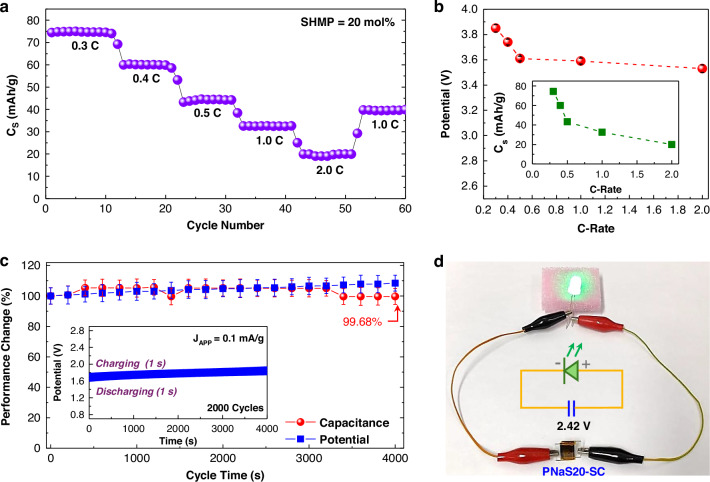
C-rate-dependent charging characteristics and endurance. **a** Specific capacity (C_S_) of supercapacitors (PNaS20-SCs) as a function of C-rate (0.3, 0.4, 0.5, 1, and 2 C), where each C-rate includes 10 charge-discharge cycles conducted over different time intervals. **b** Device potential (inset: Cs) as a function of C-rate. **c** Capacitance and potential variations over repeated charge-discharge cycles by charging for 1 s at J_APP_ = 0.1 mA/g and discharging for 1 s at J_APP_ = 0 mA/g (inset: change of device potential as a function of cycle time (s)). **d** Demonstration of operating a green LED using a single PNaS20-SC device charged to 2.42 V

To further investigate the long-term charging behaviors of PNaS-supercapacitors (SHMP = 20 mol%), a stepwise charging test was attempted by varying C-rates from 0.3 C to 2.0 C. As illustrated (Fig. 5a), the specific capacity (Cs) of devices remained stable over^10^ consecutive charge/discharge cycles at each C-rate, indicating good charging/discharging fidelity. A maximum Cs of 75.0 mAh/g was recorded at a C-rate of 0.3 C, which is comparable to the performance of conventional graphite-based lithium supercapacitors^58^. In detail, by increasing the C-rate from 0.3 to 2.0 C, the specific capacity gradually decreased from 75.0 to 11.3 mAh/g. This reduction is attributed to the limited sodium ion transport capability within the PNaS SSE (SHMP = 20 mol%) under high current densities, as typically observed in conventional rechargeable (lithium-ion) battery systems^59^. Importantly, as the C-rate was reduced from 2.0 to 1.0 C, the specific capacity could recover from 19.0 to 39.87 mAh/g. This indicates that the present PNaS-supercapacitors exhibit excellent reversibility and stability in charging/discharging performance under various operation conditions. As plotted in Fig. 5b, the resulting potential initially showed a noticeable reduction from 3.85 V at 0.3 C to 3.6 V at 0.5 C, followed by a very slow change to 3.53 V at 2.0 C. In brief, despite a 660% increase in C-rate (from 0.3 C to 2.0 C), the potential drop was only 8.3% (from 3.85 V to 3.53 V), whereas a typical large Cs variation was measured with the C-rate (Fig. 5b inset).

Based on these promising C-rate-dependent results, endurance tests were conducted on the PNaS-supercapacitors by applying 2000 charging/discharging cycles at J_APP_ = 0.1 mA/g. As shown in Fig. 5c, the device potential exhibited only a slight variation (ca. 1%) over 2000 cycles (see inset graph for time-dependent potential changes in Fig. 5c), and the capacitance was well-maintained at 99.68% of the initial value (note that the present devices exhibited excellent stability upon 2000 cycles in the wide potential range of 0 – 4 V - see Supplementary Fig. 11). This minimal loss (0.32%) of capacitance confirms the excellent long-term cycling stability and robust sodium-ion transport within the PNaS SSEs (SHMP = 20 mol%), positioning these devices among the most stable graphite-based supercapacitors reported to date^60^. For actual applications, the PNaS-supercapacitors were tested to operate light-emitting diodes (LEDs). As shown in Fig. 5 d, a single PNaS-supercapacitor, which was charged to ca. 4.4 V at J_APP_ = 0.5 mA/g for 10 s, could successfully operate green and yellow LEDs at a stable voltage of 2.42 V after connection (see video clips in Supplementary Fig. 12). Finally, a brief flammability test was conducted by directly contacting a lighter fire (flame) to the PNa and PNaS films coated on glass substrates. As shown in Supplementary Fig. 13a, b, the PNa film was easily burned and damaged, resulting in a noticeable change in film shape, upon direct contact with the flame. In contrast, the PNaS film did show only minimal color change (by burning) and retained its film shape well (see the final shapes in Supplementary Fig. 13c). Note that the PNa film emitted a strong smell like ammonia or amine derivatives upon the flame test, which can be attributed to the thermal degradation of nitrogen groups in bPEI. However, almost no smell was felt from the PNaS film during the test, supporting good flame-retardant characteristics. This result reflects that SHMP might play a key role in suppressing the decomposition of bPEI chains due to the nonflammable phosphorus elements in the SHMP molecules^61,62^. Finally, the PNaS-supercapacitors were evaluated at elevated temperatures up to 110 °C. As demonstrated in Supplementary Fig. 14, the potential of devices reached ca. 3 V at 70 ^o^C but noticeably decreased to ca. 1.6 V at 90 ^o^C. Even after quick discharging by the ESD effect, the device potential was still maintained in the range of 1.7 V (70 ^o^C) and 1.2 V (110 ^o^C). This result supports that the present PNaS20-supercapacitors can be used at temperatures up to 110 ^o^C when variable potentials are permitted for applications. Here, it is noted that additional mechanical endurance tests are necessary for practical applications, as previously reported on impact-resistant batteries and supercapacitors^63–65^.

## Conclusions

A water-processable sodium-based solid-state electrolyte (bPEI:NaOH:SHMP - PNaS) was successfully prepared by combining the water-soluble three components with various SHMP molar ratios and applied to sodium-ion supercapacitors with a single active material electrode configuration. The resulting PNaS SSEs were optically clear without any noticeable color, even though the SHMP molar ratio was varied up to 40 mol%, which led to well-aligned electrolyte layers in the device structure of glass/ITO/GSP/PNaS/ITO/glass. The EIS measurements showed that the ion conductivity of PNaS SSEs was strongly dependent on the SHMP molar ratio, leading to ~1 mS/cm at SHMP = 20 mol% (compared to 0.18 mS/cm at SHMP = 0 mol%) due to the formation of the SHMP micro-domains, enabling faster transport of sodium cations under a reduced physical impedance (caused by the hydrophobic part of bPEI chains). Galvanostatic charge-discharge (GCD) measurements revealed that the PNaS-supercapacitors (SHMP = 20 mol%) could achieve the highest device potential and energy density (4.1 V and 15 mWh/g at J_APP_ = 0.5 mA/g), compared to 2.1 V and 6.9 mWh/g at SHMP = 0 mol%, and delivered the highest potential level retained even after natural discharge conditions (ca. 1.0 V after 1000 s). The C-rate test confirmed that the PNaS-supercapacitors (SHMP = 20 mol%) could operate excellently under different charging/discharging rates (only 8.3% potential decrease despite 660% C-rate increase). The endurance test revealed that the PNaS-supercapacitors exhibited an outstanding capacitance retention of 99.68% even after 2000 charge/discharge cycles. The optimized PNaS-supercapacitors could stably drive both green and yellow LEDs repeatedly, while a brief flame test confirmed outstanding nonflammability for the PNaS films. These results strongly support the viability and practicability of the present water-processable PNaS SSEs and the promising future of sodium-based solid-state electrolytes for energy storage devices.

## Methods

### Materials, solutions, and compounds

Sodium hexametaphosphate (SHMP, 65-70% P₂O₅ basis), branched poly(ethylene imine) (bPEI, aqueous solution, ~1 wt%, weight-average molecular weight of ~25 kDa, number-average molecular weight of ~10 kDa, polydispersity index of ~2.5), poly(vinylidene fluoride) (PVdF, weight-average molecular weight of ~534 kDa), and N-methyl-2-pyrrolidone (NMP) were purchased from Sigma-Aldrich (USA) and used as received without further purification. Sodium hydroxide (NaOH, EP Grade, pellet form) was obtained from Duksan (Republic of Korea). Super P-Li (density = 1.60 g/cm³, BET surface area 62.0 m²/g), and ultra-fine artificial graphite powder (average particle diameter = ~30 µm) were supplied from TIMCAL (Switzerland) and SHOWA DENKO (Japan), respectively. Binary solutions of bPEI and NaOH were prepared by dissolving the respective amounts of each component in 1 mL of deionized water and mixing thoroughly. Subsequently, fully dissolved SHMP solutions were added at the SHMP molar ratio of 0, 10, 20, 30, and 40 mol% relative to the repeating units of bPEI. The exact weight ratios of bPEI, NaOH, and SHMP in the PNaS solutions, with a total volume of 3 mL of deionized water, were 0.533 g:0.04 g:0 g (0 mol%), 0.533 g:0.04 g:0.0772 g (10 mol%), 0.533 g:0.04 g:0.1545 g (20 mol%), 0.533 g:0.04 g:0.3089 g (30 mol%), and 0.533 g:0.04 g:0.4634 g (40 mol%). These ternary PNaS mixtures were stirred at 60 °C for 15 h prior to film coating processes. To prepare the GSP pastes for anode layers used in supercapacitors, graphite (9.0 g), Super P-Li (0.3 g), and PVdF (0.7 g) were mixed in NMP at a concentration of 10 mg/mL.

### Fabrication of supercapacitors

To fabricate asymmetric supercapacitors with single active-material electrodes, indium tin oxide (ITO)-coated glass substrates were first patterned to make a sheet of ITO electrodes (12 mm × 8 mm) with a sheet resistance of 10 Ω/cm². The patterned substrates were then thoroughly cleaned using acetone and isopropyl alcohol, dried under argon flow, and treated with a UV-ozone (UVO) cleaner (AC-6, AHTECH LTS Co., Ltd.) at an intensity of 50 mW/cm² for 20 min. Subsequently, GSP layers (~150 µm thick) were deposited onto the UVO-treated ITO-glass substrates and dried in a muffle furnace at 100 °C for 15 h. Next, the PNaS electrolyte solutions were drop-cast onto the GSP layers and soft-baked at 60 °C for 15 min to form the PNaS solid-state electrolyte (SSE) films. For comparison, identical PNaS SSE films were also directly coated onto the UVO-treated ITO-glass substrates without GSP layers. Then, 150 µm-thick polyimide (PI) film spacers were placed along the substrate edges to maintain consistent film thickness. Finally, the PNaS SSE film-coated ITO-glass substrates were stacked onto the PNaS SSE film-coated GSP/ITO-glass substrates, resulting in asymmetric supercapacitors (glass/ITO/GSP/PNaS/ITO/glass).

### Measurements and analysis

Film thickness was measured using a surface profilometer (Dektak XT, Bruker) and validated by cross-sectional imaging with a field-emission scanning electron microscope (FE-SEM, S-4800, Hitachi). The surface morphology of the films was also examined using the same FE-SEM system. The chemical environment of core-level atoms within the films was analyzed using an X-ray photoelectron spectroscopy system (XPS, ESCALAB 250Xi, Thermo Scientific). A Raman spectroscopy system (RENISHAW, inVia Reflex model) was used to investigate the vibrational characteristics of the film samples. A potentiostat (VersaSTAT4, AMETEK) system was employed to measure cyclic voltammetry (CV) curves, galvanostatic charge/discharge (GCD) data, and electrochemical impedance spectroscopy (EIS) data. The EIS spectra were measured by sweeping the frequency from 100 kHz to 100 mHz. The ion conductivity (*σ*) of films was calculated using σ **=** *d* / (*R* × *A*), where *d* is the film thickness, *R* is the charge transfer resistance obtained from the real axis intercept of the Nyquist plot, and *A* is the device’s active area. The energy density (*E*) was calculated using *E* **=** 0.5·*Cs*·∆*V*^2^, where *Cs* and ∆*V* denote the specific capacitance and the potential difference from GCD data, respectively (note that the net energy density was calculated using the net potential difference without the ESD component). The flammability test was performed using a lighter flame that directly contacts the films coated on glass substrates in an air ambient condition.

## Supplementary information

Supporting Information File

Video Clip for Supplementary Figure 12a

Video Clip for Supplementary Figure 12b

Video Clip for Supplementary Figure 12c

Video Clip for Supplementary Figure 13a

Video Clip for Supplementary Figure 13b

Video Clip for Supplementary Figure 14c

## Data Availability

All data generated or analysed during the current study are included in this article and its Supplementary Information file.
