# Supplementary material for: High-retention sodium supercapacitors with sodium hexametaphosphate-controlled water-processable/non-flammable sodium-ion solid-state electrolytes
Source: Microsyst Nanoeng. 2026 Feb 11;12:58. doi: 10.1038/s41378-026-01191-7 (PMC12894708; doi:10.1038/s41378-026-01191-7)
Supplement: Supplementary file 1 — Supporting Information File [file 41378_2026_1191_MOESM1_ESM.pdf]

# Supplementary information

## High-Retention Sodium Supercapacitors with Sodium Hexametaphosphate-Controlled Water-Processable/Non-flammable Sodium-Ion Solid-State Electrolytes

Deepu Murukadas[1,2], Dahyeon Park[1,3], Minjae Kim[1,3], Hwajeong Kim[1,4], Youngkyoo Kim[1,2,3,*]

[1]Organic Nanoelectronics Laboratory and KNU Institute for Nanophotonics Applications (KINPA), Department of Chemical Engineering, School of Chemical Engineering and Applied Chemistry, Kyungpook National University, Daegu 41566, Republic of Korea

[2]Department of Energy Convergence & Climate Change and the Institute for Global Climate Change and Energy, Kyungpook National University, Daegu 41566, Republic of Korea

[3]School of Semiconductor Convergence Engineering, Kyungpook National University, Daegu 41566, Republic of Korea

[4]Priority Research Center, Research Institute of Environmental Science & Technology, Kyungpook National University, Daegu 41566, Republic of Korea

*Corresponding Author: Prof. Y. Kim

E-mail) ykimm@knu.ac.kr; Tel) +82-(0)53-950-5616

**Supplementary Table 1.** Summary of previous works on water-soluble polymer-based solid-state electrolytes for supercapacitor applications.

| No. | Water-Soluble Polymer | Salt | Electrolyte Composition | Ion conductivity (mS/cm) | Tested Cycle | Hazardous Issue | Max. Potential (Applied Current) | Ref. (Year) |
|---|---|---|---|---|---|---|---|---|
| 1 | Poly(vinyl alcohol) | LiTFSI (40 wt%) | Polymer/Salt/EMITFSI | 3.6 | 1000 | Halogen | 2.0 V (400 mA/g) | SR-1 (2018) |
| 2 | Poly(vinyl alcohol) | NaTf (40 wt%) | Polymer/Salt | 2.31 | 1000 | Halogen | 1.0 V (200 mA/g) | SR-2 (2019) |
| 3 | Poly(ethylene oxide) | $LiBF_4$ (5 mol%) | Polymer blend/Salt | 0.2(RT) | 1000 | Halogen | 0.5 V (1000 mA/g) | SR-3 (2019) |
| 4 | Poly(ethylene oxide) | $LiClO_4$ (40 wt%) | Polymer blend/Salt | 0.734 | 100 | Halogen | 1.0 V (0.5 $mA/cm^2$) | SR-4 (2019) |
| 5 | Poly(vinyl alcohol) + chitosan | $LiClO_4$ (40 wt%) | Polymer/Salt | 0.845 | 100 | Halogen | 0.9 V (0.5 $mA/cm^2$) | SR-5 (2020) |
| 6 | Corn starch | LiOAc (24 wt%) | Polymer/Salt/$TiO_2$ | 0.837 | - | No | 1.9 V (0. $mA/cm^2$) | SR-6 (2021) |
| 7 | PEO/$Na_3Zr_2Si_2PO_{12}$/NaI | NaI (10 wt%) | Polymer/Salt | 0.1 | 400 | Halogen | 2.0 V (2000 mA/g) | SR-7 (2021) |
| 8 | PVA/PVP | NaSCN (20 wt%) | Polymer blend/Salt | 0.81 | - | No | 1.5 V (500 mA/g) | SR-8 (2022) |
| 9 | Poly(ethylene oxide) | LiBr (4 mol%) | Polymer blend/Salt | 0.375 | 500 | Halogen | 0.375 | SR-9 (2023) |
| 10 | Branched-poly(ethylene imine) (bPEI) | LiOH (27 wt%) | Polymer/Salt | ~1 | 700 | No | 0.9 V (0.4 mA/g) | SR-10 (2023) |
| 11 | Branched-poly(ethylene imine) (bPEI) | LiOH (27 wt%) | Polymer/Salt/ZnO | 1.36 | 1000 | No | 2.0 V (0.4 mA/g) | SR-11 (2024) |
| 12 | Branched-poly(ethylene imine) (bPEI) | LiOH (55.5 wt%) | Polymer/Salt/PSSA | 0.8116 | 5000 | No | 2.24 V (0.2 mA/g) | SR-12 (2024) |
| 13 | Poly(vinyl alcohol) | LiBr (250 mol%) | Polymer /Dual Salt | ~10 | 1700 | Halogen | 2.1 V (5 A/g) | SR-13 (2025) |
| 14 | Branched-poly(ethylene imine) (bPEI) | NaOH (6 wt%) | Polymer/Salt/SHMP | ~1 | 2000 | No | 4.1 V (0.5 mA/g) | This Work 2025 |

## < References for Supplementary Table 1 >


SR-1. Wang, J., Zhao, Z., Song, S., Ma, Q. & Liu, R. High performance poly (vinyl alcohol)-based Li-ion conducting gel polymer electrolyte films for electric double-layer capacitors. *Polymers* **10**, 1179 (2018).

SR-2. Farah, N. *et al.* Solid polymer electrolytes based on poly (vinyl alcohol) incorporated with sodium salt and ionic liquid for electrical double layer capacitor. *Mater. Sci. Eng. B-Adv. Funct. Solid-State Mater.* **251**, 114468 (2019).

SR-3. Karaman, B., Çevik, E. & Bozkurt, A. Novel flexible Li-doped PEO/copolymer electrolytes for supercapacitor application. *Ionics* **25**, 1773–1781 (2019).

SR-4. Aziz, S. B. *et al.* Fabrication of energy storage EDLC device based on CS: PEO polymer blend electrolytes with high Li+ ion transference number. *Results Phys.* **15**, 102584 (2019).

SR-5. Brza, M. *et al.* Energy storage behavior of lithium-ion conducting poly (vinyl alcohol)(PVA): Chitosan (CS)-based polymer blend electrolyte membranes: Preparation, equivalent circuit modeling, ion transport parameters, and dielectric properties. *Membranes* **10**, 381 (2020).

SR-6. Ong, A. C. W., Shamsuri, N. A., Zaine, S. N. A., Panuh, D. & Shukur, M. F. Nanocomposite polymer electrolytes comprising starch-lithium acetate and titania for all-solid-state supercapacitor. *Ionics* **27**, 853–865 (2021).

SR-7. Singh, M. D., Kaur, G., Sharma, S. & Dalvi, A. All-solid-state Na+ ion supercapacitors using $Na_3Zr_2Si_2PO_{12}$-polymer hybrid films as electrolyte. *J. Energy Storage* **41**, 102984 (2021).

SR-8. Badi, N. *et al.* Fabrication and characterization of flexible solid polymers electrolytes for supercapacitor application. *Polymers* **14**, 3837 (2022).

SR-9. Shenbagavalli, S., Muthuvinayagam, M. & Revathy, M. S. Characterization of lithium-based poly (ethylene oxide)/poly (vinylidene fluoride-co-hexafluoropropylene) solid blend polymer electrolytes for energy storage applications. *Ionics* **29**, 211–231 (2023).

SR-10. Cho, Y., Lee, S., Kim, H. & Kim, Y. Lithium polymer supercapacitors with water-processable branched poly (ethylene imine)-based solid-state electrolytes. *J. Energy Storage* **57**, 106010 (2023).

SR-11. Murukadas, D., Cho, Y., Lee, W., Lee, S., Kim, H. & Kim, Y. Lithium supercapacitors with environmentally friendly water-processable solid-state hybrid electrolytes of zinc oxide/polymer/lithium hydroxide. *Energy* **290**, 129984 (2024).

SR-12. Murukadas, D., Kim, H. & Kim, Y. Pronounced role of lithium controlling polymer in water processable/halogen-free all solid state electrolytes for lithium supercapacitors. *Adv. Sci.* **12**, 2417745 (2025).

SR-13. Ismail, A. A., Khedr, G. E., Akar, A. A., Ghanem, L. G. & Allam, N. K. Solid acid–dual salt hybrid electrolyte unlocks broad electrochemical stability and high capacitance in solid-state devices. *ACS Appl. Mater. Interfaces* **17**, (2025).

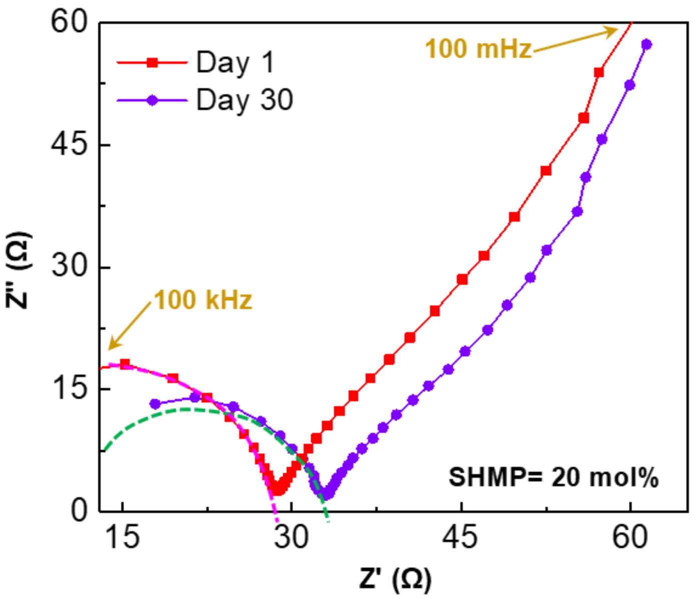


**Supplementary Fig. 1** | Nyquist plots of glass/ITO/GSP/PNaS20/ITO/glass devices (SHMP = 20 mol%) measured just after fabrication (day 1) and after storing for 30 days. Note that the starting and ending frequencies were 100 mHz and 100 kHz, respectively. The ion conductivity was marginally reduced from 1 mS/cm (day 1) to 0.82 mS/cm (day 30).

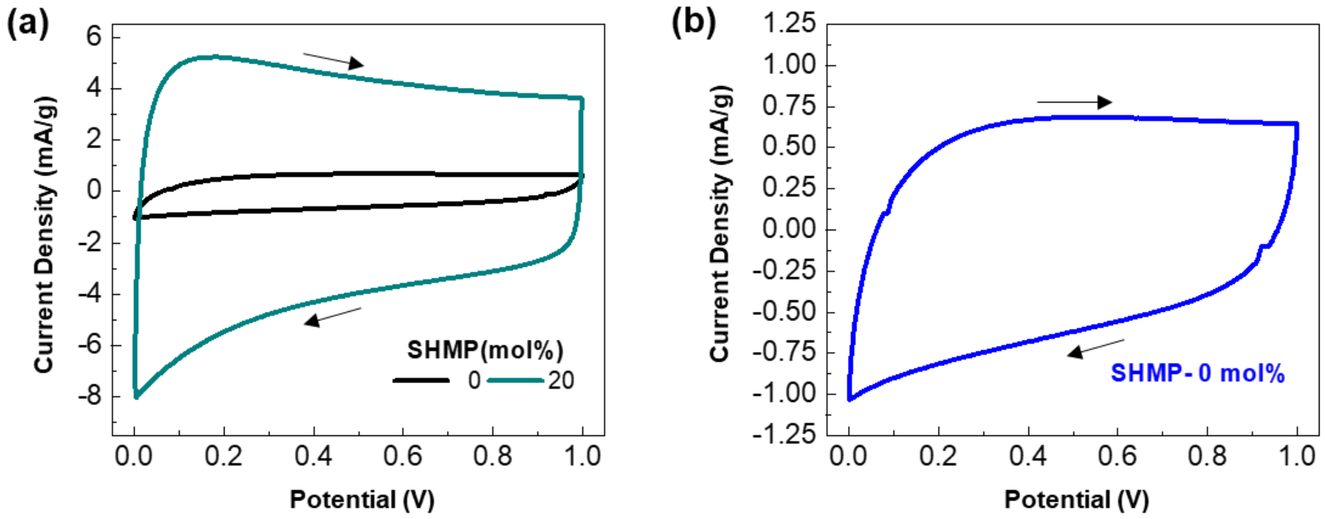


**Supplementary Fig. 2** | Cyclic voltammetry (CV) curves of the supercapacitors with the PNaS SSEs at a sweep rate of 1.0 V/s (arrows denote the direction of potential sweeps): **a,** Comparison between SHMP = 0 mol% and SHMP = 20 mol%. **b,** Enlarged CV for SHMP = 0 mol%.

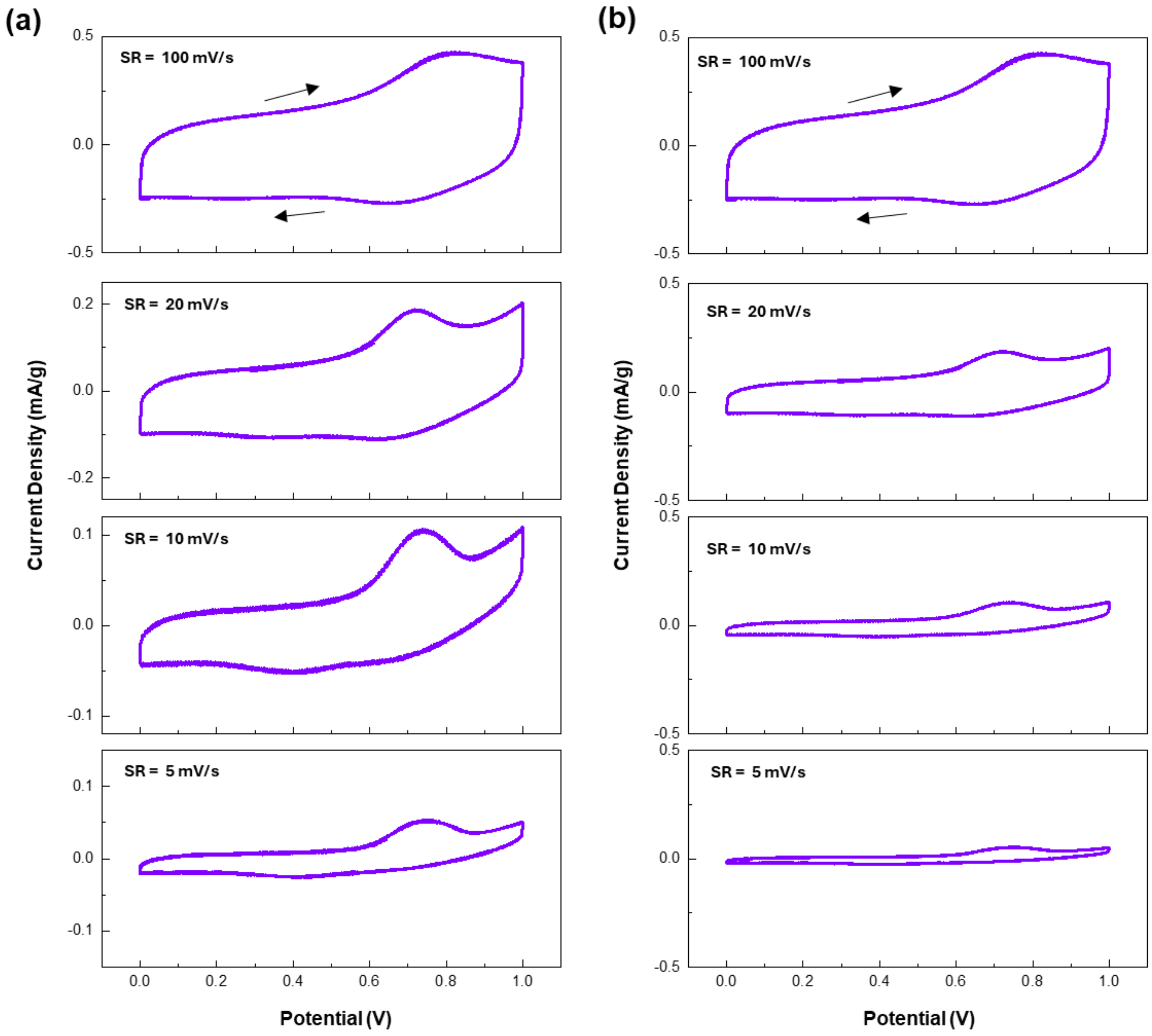


**Supplementary Fig. 3 |** Cyclic voltammetry (CV) curves of the supercapacitors with the PNaS SSEs (SHMP = 20 wt%) at the narrow potential window of 0 V and 1.0 V (see black arrows for the direction of potential sweep). The sweep rates (SRs) applied were 100 mV/s, 20 mV/s, 10 mV/s, and 5 mV/s. Note that the scale of current density was differently applied to (a) and (b) for detailed comparison.

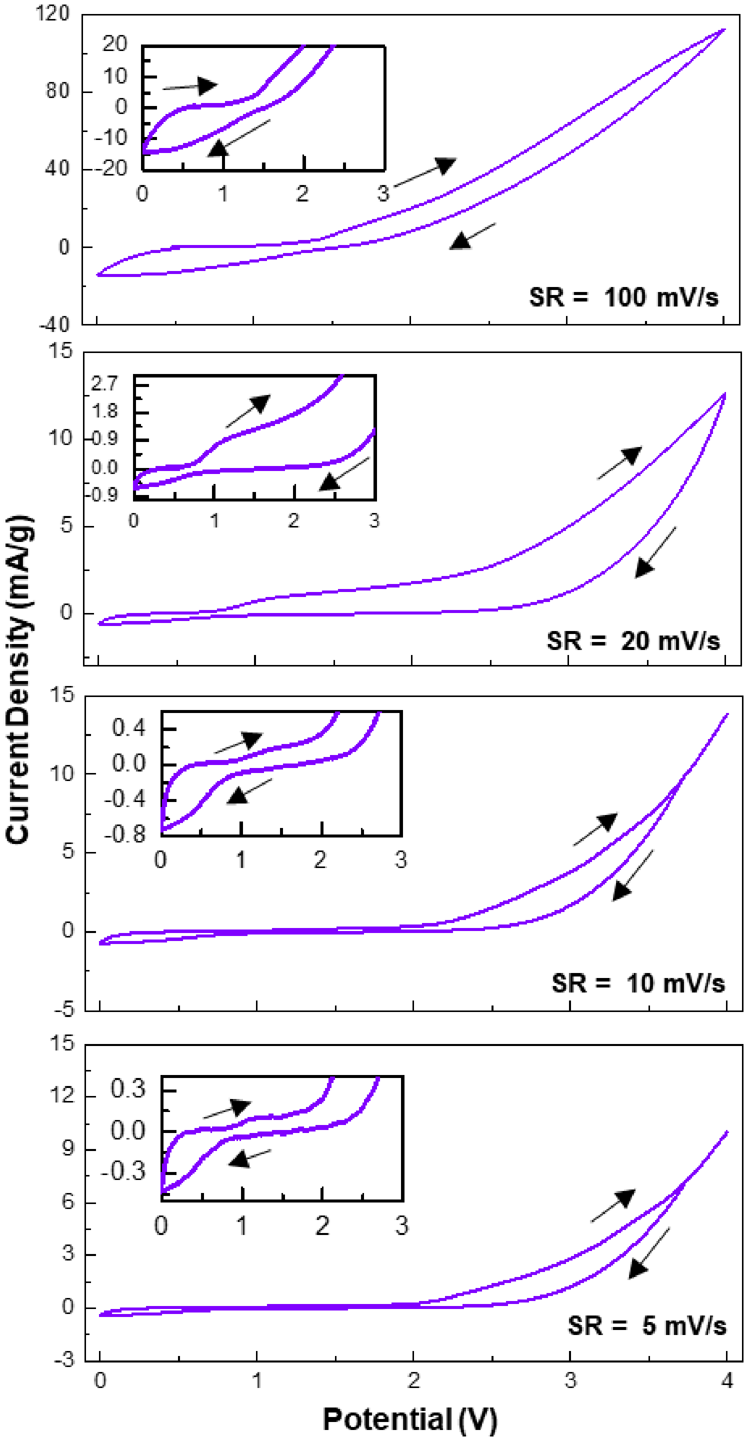


**Supplementary Fig. 4 |** Cyclic voltammetry (CV) curves of the supercapacitors with the PNaS SSEs (SHMP = 20 wt%) at the wide potential window of 0 V and 4.0 V (see black arrows for the direction of potential sweep). The sweep rates (SRs) applied were 100 mV/s, 20 mV/s, 10 mV/s, and 5 mV/s. Inset graphs show the enlarged parts of the CV curves.

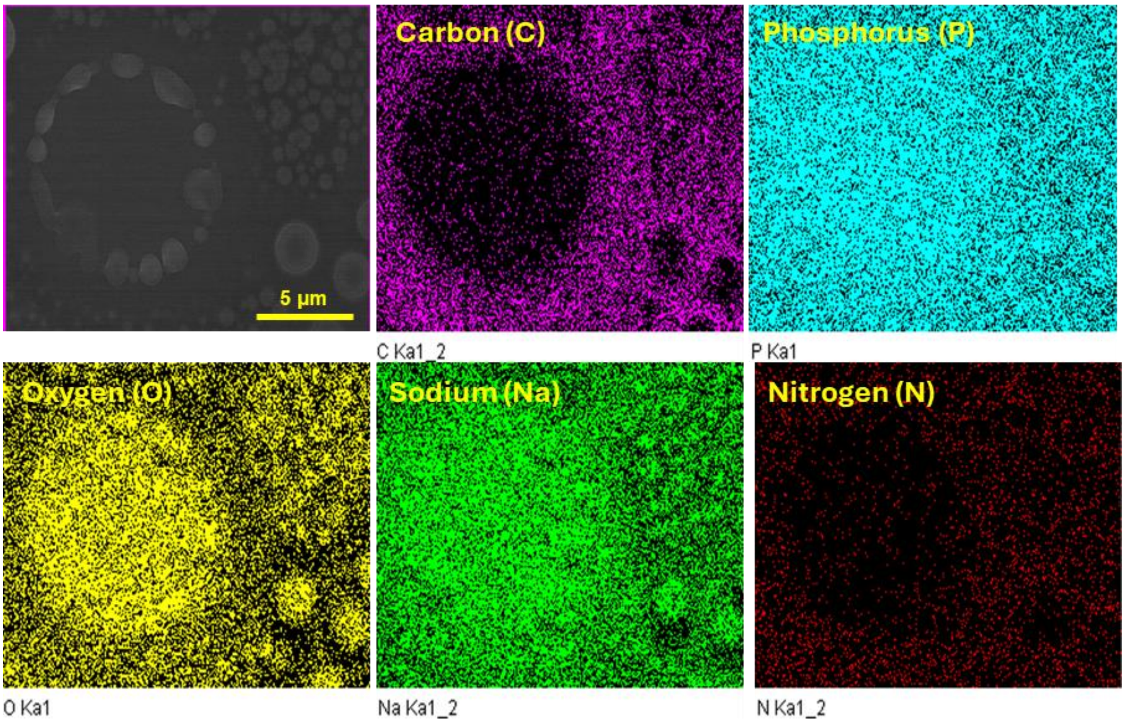


**Supplementary Fig. 5 |** Energy dispersive spectroscopy (EDS) for the PNaS SSE films: (a) carbon, (b) phosphorus, (c) oxygen, (d) sodium, and (e) nitrogen. Note that the circular micro-domain area indicates the presence of SHMP content.

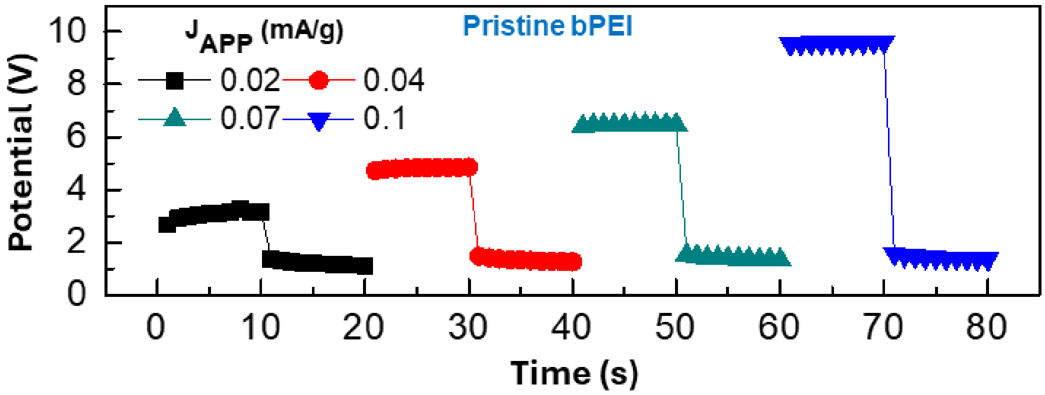


**Supplementary Fig. 6 |** Galvanostatic charge–discharge (GCD) curves for the devices with the pristine bPEI layers upon a stepwise increase of applied current density ($J_{APP}$ = 0.02, 0.04, 0.07, and 0.1 mA/g).

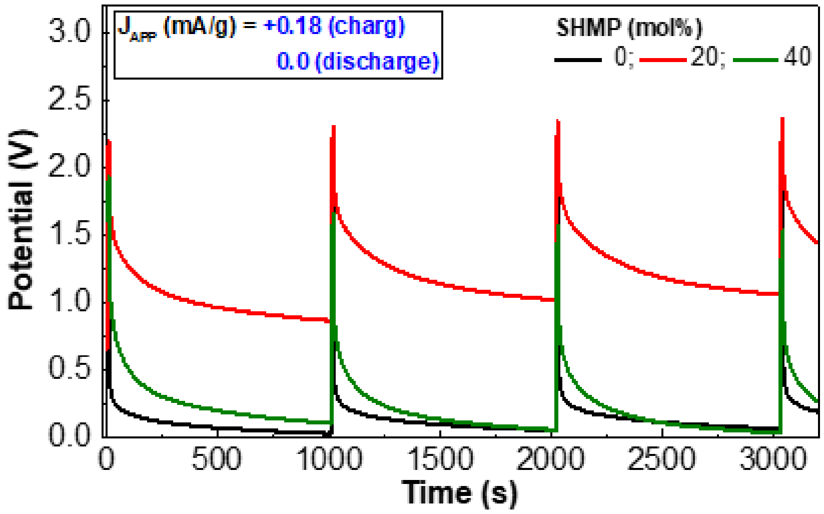


**Supplementary Fig. 7 |** Long-term discharge behavior for the three representative SHMP molar ratios under repeated operations (natural discharge for 1000 s at $J_{APP}$ = 0 mA/g after charging for 10 s at $J_{APP}$ = 0.18 mA/g). Note that the device's potential was measured quite stably despite repeated long-term charging/discharging operations.

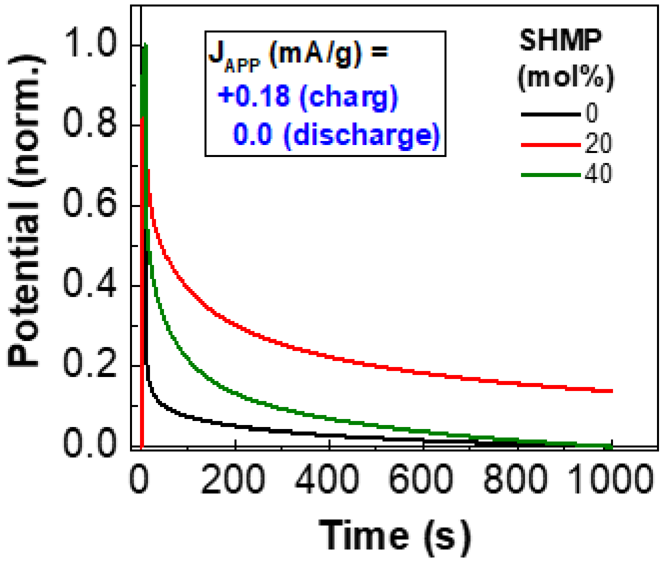


**Supplementary Fig. 8 |** Normalized long-term discharge potential for the three representative SHMP molar ratios under repeated operations (natural discharge for 1000 s at $J_{APP}$ = 0 mA/g after charging for 10 s at $J_{APP}$ = 0.18 mA/g).

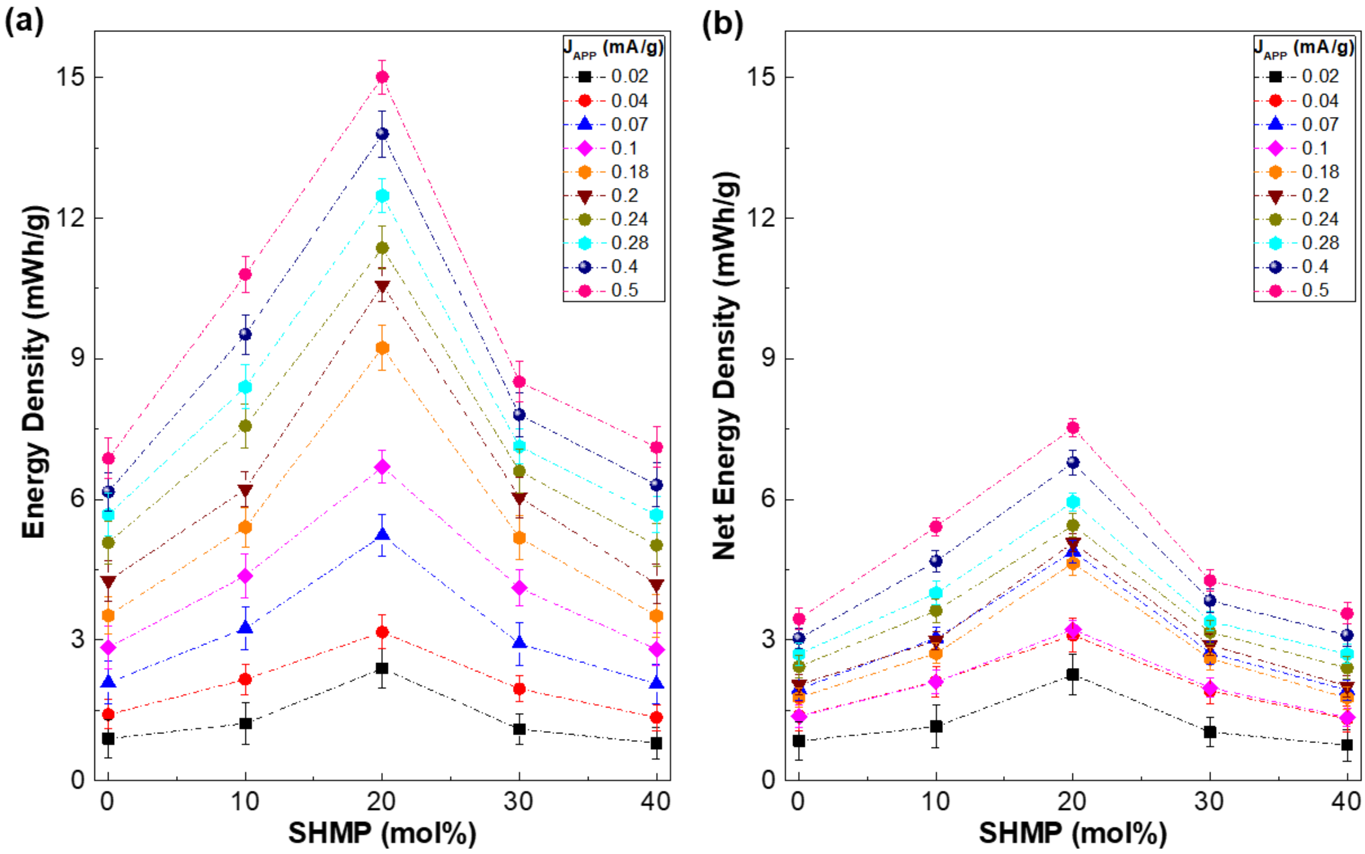


**Supplementary Fig. 9** | Energy density (ED) as a function of SHMP molar ratio for the sodium supercapacitors with the PNaS SSEs: **a,** Apparent ED calculated using the as-measured potentials; **b,** Net ED calculated using the net potentials after subtracting the ESD-induced potentials.

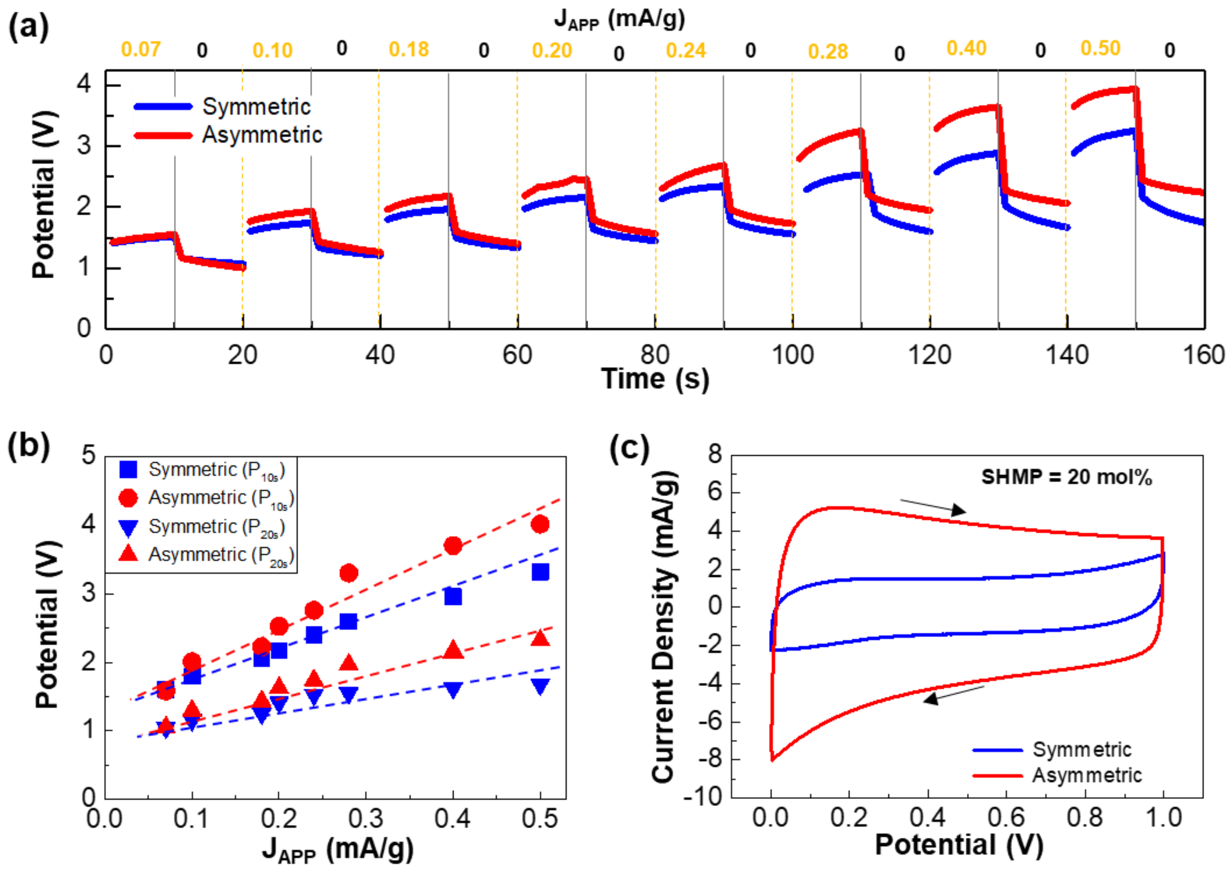


**Supplementary Fig. 10 |** Comparion of symmetric (glass/ITO/GSP/PNaS20/GSP/ITO/glass) and asymmetric (glass/ITO/PNaS20/GSP/ITO/glass) type PNaS20 supercapacitors: **a,** Galvanostatic charge–discharge (GCD) curves under the stepwise increase of applied current density ($J_{APP}$) for charging (10 s each) and natural discharging ($J_{APP}$ = 0 mA/g). **b,** Trend of potentials at 10s (maximum voltage by charging) and 20s (retained voltage after discharge for 10 s) (data taken from the GCD curves in **a**). **c,** Cyclic voltammetry (CV) curves for the symmetric and asymmetric PNaS20 supercapacitor at a sweep rate of 1.0 V/s.

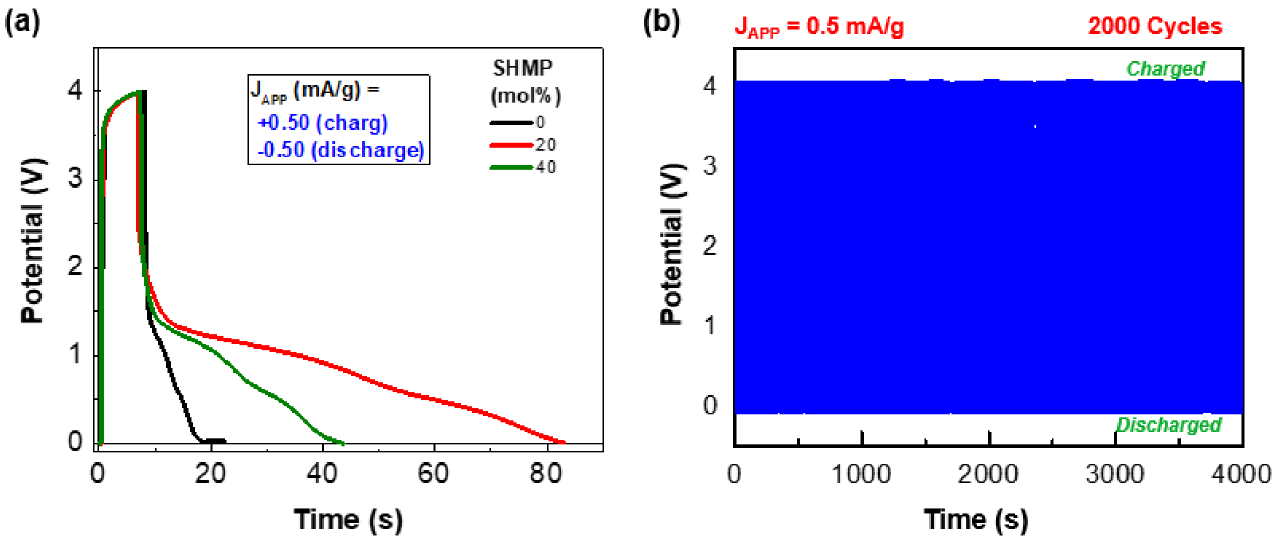


**Supplementary Fig. 11** | Charging/discharging characteristics for the PNaS20 supercapacitors: **a,** GCD curves under forced charge/discharge conditions by limiting the maximum potential to 4.0 V (charging at $J_{APP}$ = +0.5 mA/g; discharging at $J_{APP}$ = -0.5 mA/g). **b,** Change of device potential as a function of charge/discharge cycle time (s) for the PNaS20 supercapacitors (2000 cycles).

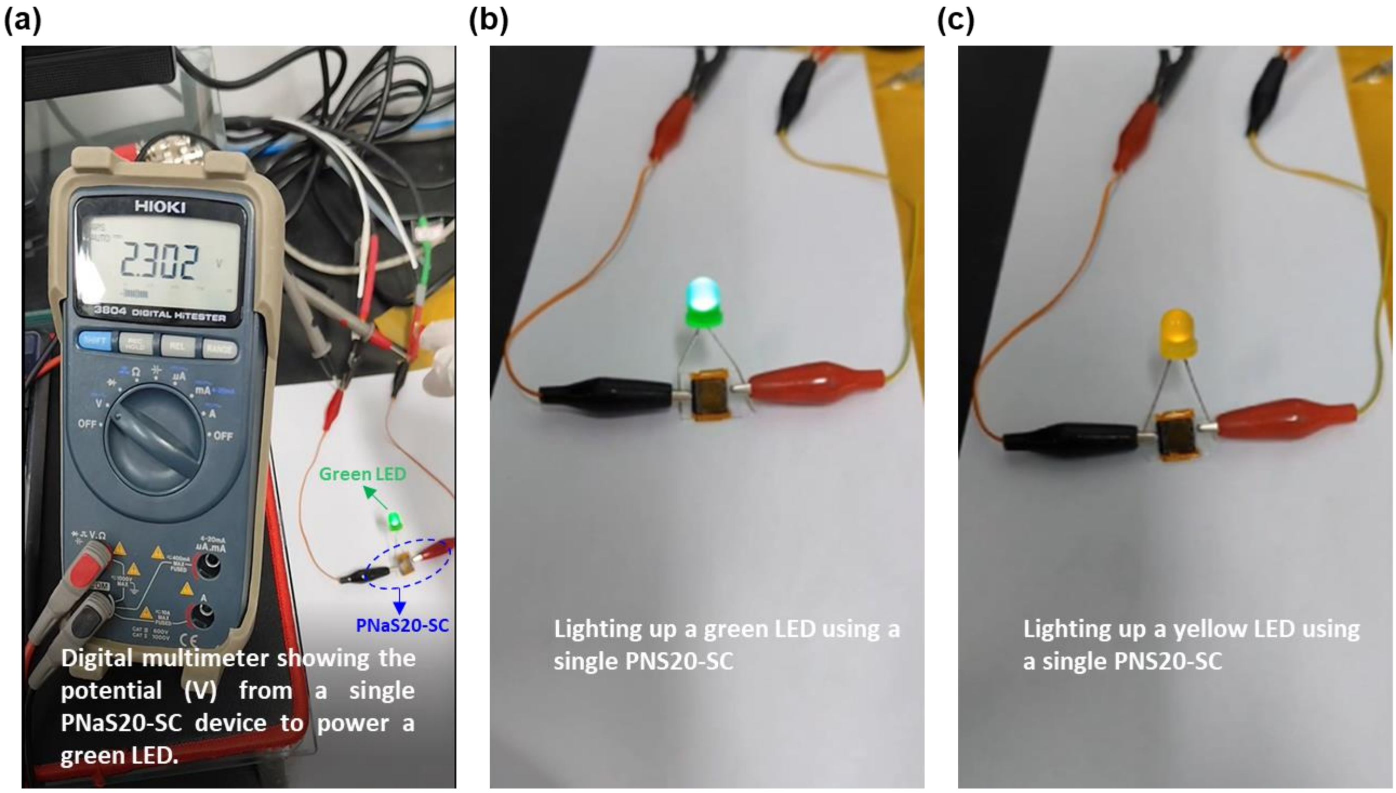


**Supplementary Fig. 12 |** Demonstration of LED operation using PNaS-based supercapacitors: **a,** A digital multimeter displaying the voltage output from a single PNaS20-SC device connected to a green LED emitting light. **b,c** Operation of green and yellow LEDs using a single PNaS20-SC device charged (see video clips: Fig_S12a_video.mp4, Fig_S12b_video.mp4, Fig_S12c_video.mp4).

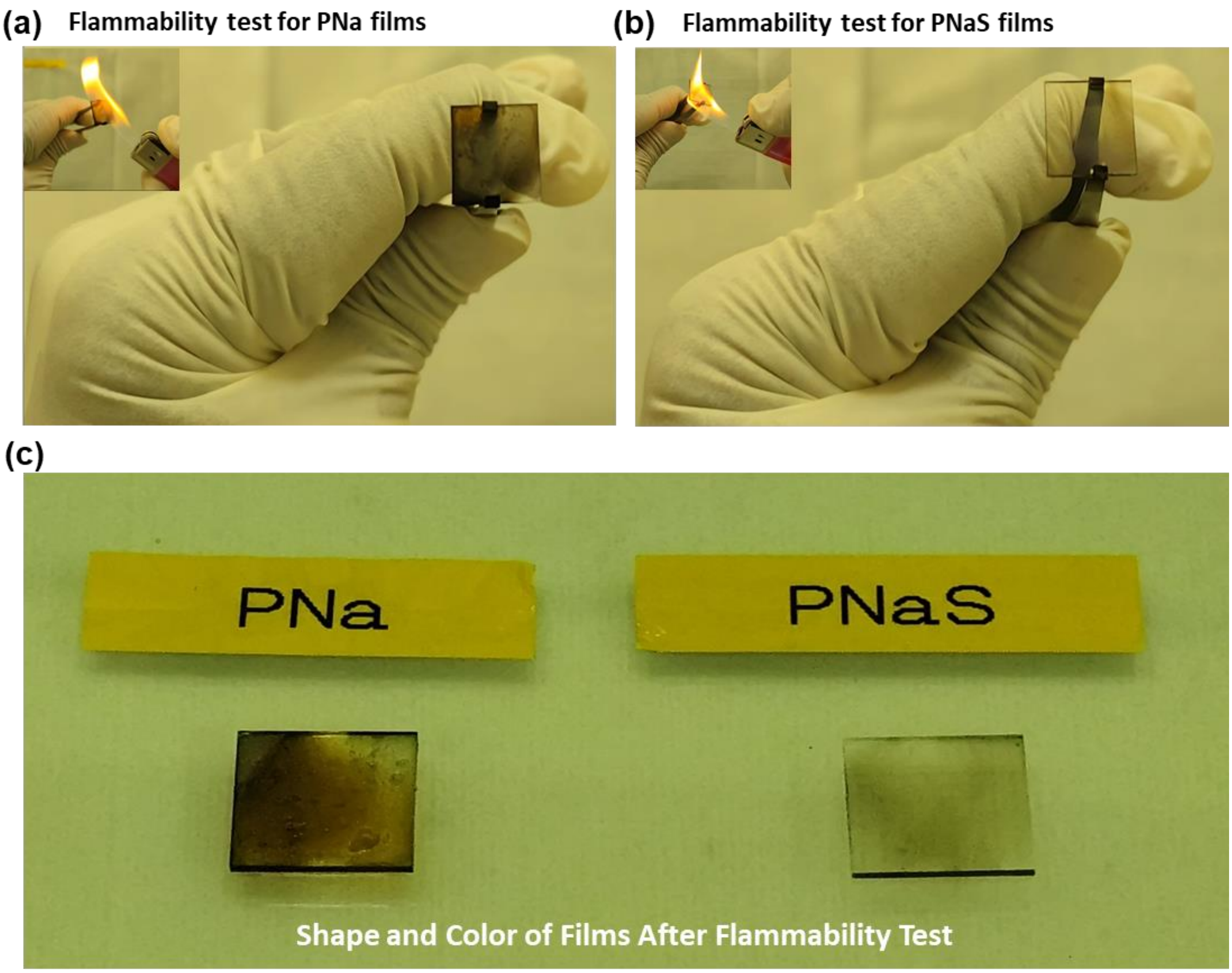


**Supplementary Fig. 13 |** Flammability tests for PNa and PNaS thin-films: **a,b,** Photographs of PNa and PNaS thin-films coated on glass substrates after flammability test by direct exposure to a lighter flame (see inset images). **c,** Comparison between the flame-tested PNa and PNaS thin-films. Although the PNa thin-film was significantly damaged and burned by the direct contact of flame, the PNaS thin-film showed relatively small damage and very slight color change (see video clips: Fig_S13a_video.mp4, Fig_S13b_video.mp4).

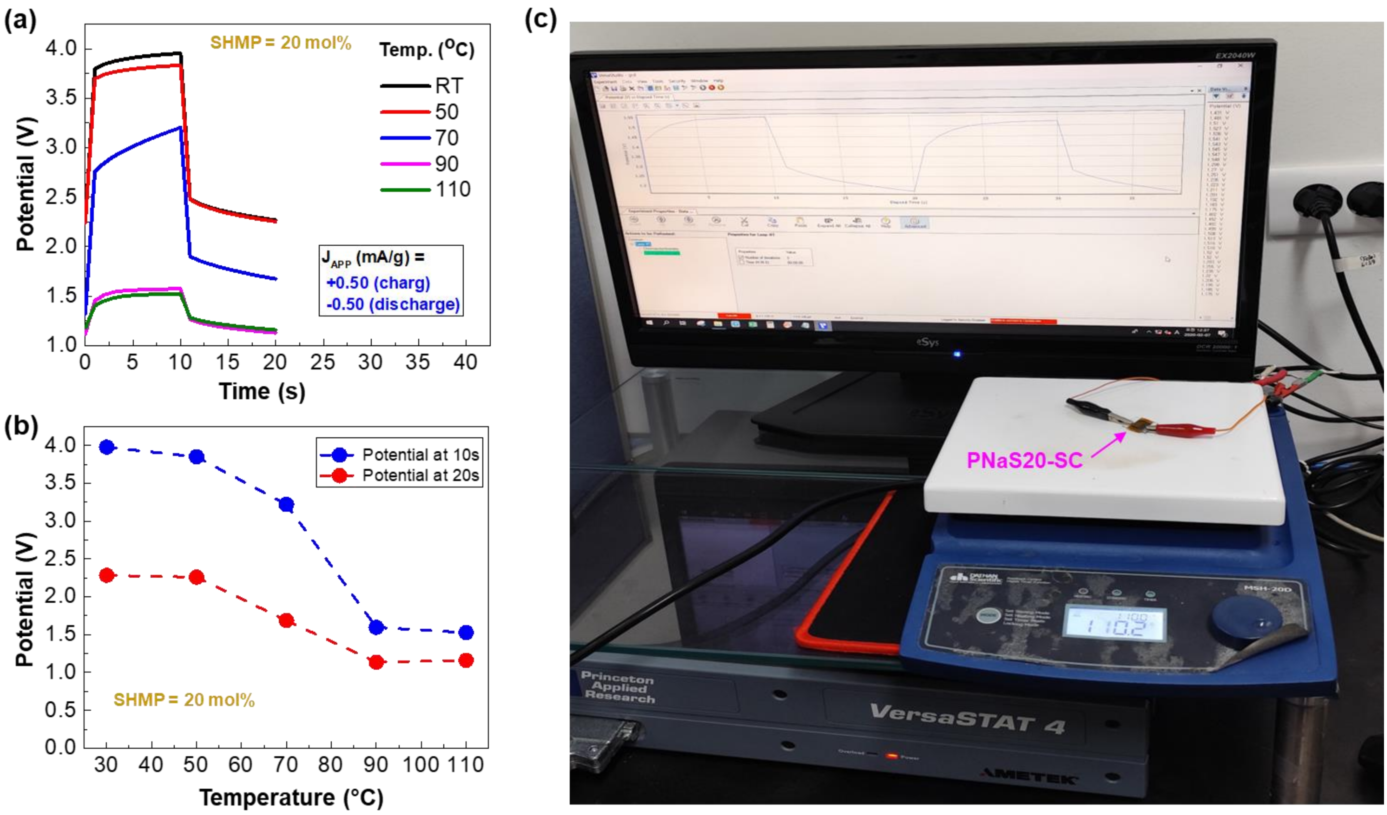


**Supplementary Fig. 14 |** Device performance of PNaS20 supercapacitors at elevated temperatures: **a,** GCD curves at RT (25 °C), 50 °C, 70 °C, 90 °C, and 110 °C (see $J_{APP}$ conditions on the graph). **b,** Change of device potential as a function of temperature. **c,** Photograph for the operation of the supercapacitor (PNaS20-SC) at 110 °C (see video clips: Fig_S14c_video.mp4).
